# Supplementary material for: Early asymmetric cues triggering the dorsal/ventral gene regulatory network of the sea urchin embryo
Source: eLife. 2014 Dec 2;3:e04664. doi: 10.7554/eLife.04664 (PMC4273433; doi:10.7554/eLife.04664)
Supplement: Supplementary file 1. — List of gene specific oligonucleotides used in the quantitative RT-PCR. DOI: http://dx.doi.org/10.7554/eLife.04664.017 [file elife04664s001.doc]

**Table S1. List of gene-specific oligonucleotides used in the quantitative RT PCR.**

| Target gene | Forward (F) and Reverse (R) sequences (5’ to 3’) | Length | Amplicon size (bp) | Figures in which oligonucleotide was used |
| --- | --- | --- | --- | --- |
|  |  |  |  |  |
| *hbox12* | F: ACGTCTTCGTCGAGCATCTC  R: GCATGGTGCCTTTCGCTTACG | 20  21 | 124 | 1A |
| *nodal* | F: ACAACCCAAGCAACCACGCA  R: CGCACTCCTGTACGATCATG | 20  20 | 174 | 1A, 2H-I, 2-S1, 4C, 4-S2 |
| *gsc* | F: ACGACAACGACATCATCTCGG  R: CCGTAGCGTCCTGAAGTGAC | 21  20 | 98 | 2I, 2-S1, 4C, 4-S2 |
| *tbx2/3* | F: AACATCTCGGACAAGCACGGA  R: GGTGACGGCAATGAAGACAGT | 21  21 | 156 | 2I, 2-S1, 4C |
| *strim1* | F: CGATGAATTAAAGAACACCATGAC  R: GGTTGTCCAGGCACGCCTTAC | 24  21 | 124 | 2I, 4C |
| *otp* | F: CGGCACAACTGAACGAACTGG  R: CGAAATACATTAGTGGTCTTCTTG | 21  24 | 172 | 2I, 4C |
| *pax2/5/8* | F: CCCGACAAGGACTCTAACCAG  R: GCCTCTGACACGACGCTGCT | 21  22 | 138 | 2I, 4C |
| *fgfA* | F: TACATTTGAAGTGGTTGGATTTCG  R: GCCAGGGACACGCATAAGAAC | 24  21 | 159 | 4C |
| *gcm* | F: GCTCGTAAGAAGCAAGGTGATAA  R: TTAGATTGGAACAGGATGACAGTA | 23  24 | 143 | 4C |
| *mbf-1*^§^ | F: ATGACACAGCCTGGAGCT  R: TACCAAGGAAGTGGGTGT | 18  18 | 102 | 1A, 2H-I, 2-S1, 4C, 4-S2 |
| *cit-ox*^§^ | F: GTTGGGGTTAATCTAACATTCTTC  R: GAGGGTATAGGCATCTGGATAG | 24  22 | 93 | 1A, 2H-I, 2-S1, 4C, 4-S2 |
| *z12*^†^ | F: AGCGCCACACCAAAAGAAGTC  R: GGATGATAGACAGGGCTGTTTGGA | 21  24 | 93 | 1A, 2H-I, 2-S1, 4C, 4-S2 |

^§^ The *H2A* histone modulator binding factor (*mbf-1*), *z12*, or a *cytochrome-oxidase* (*cyt-ox*) mRNA were used to normalize all data.

^†^ The *z12* mRNA was used to estimate the number of *hbox12* and *nodal* transcripts per embryo.
